# Supplementary material for: Causal Relationship between Aging and Anorexia Nervosa: A White-Matter-Microstructure-Mediated Mendelian Randomization Analysis
Source: Biomedicines. 2024 Aug 16;12(8):1874. doi: 10.3390/biomedicines12081874 (PMC11351342; doi:10.3390/biomedicines12081874)
Supplement: Supplementary file 1 [file biomedicines-12-01874-s001.zip › Supplementary Figure/Figure S4/Singe SNP plot of MR analysis for TL to ubm-b-1712.pdf]

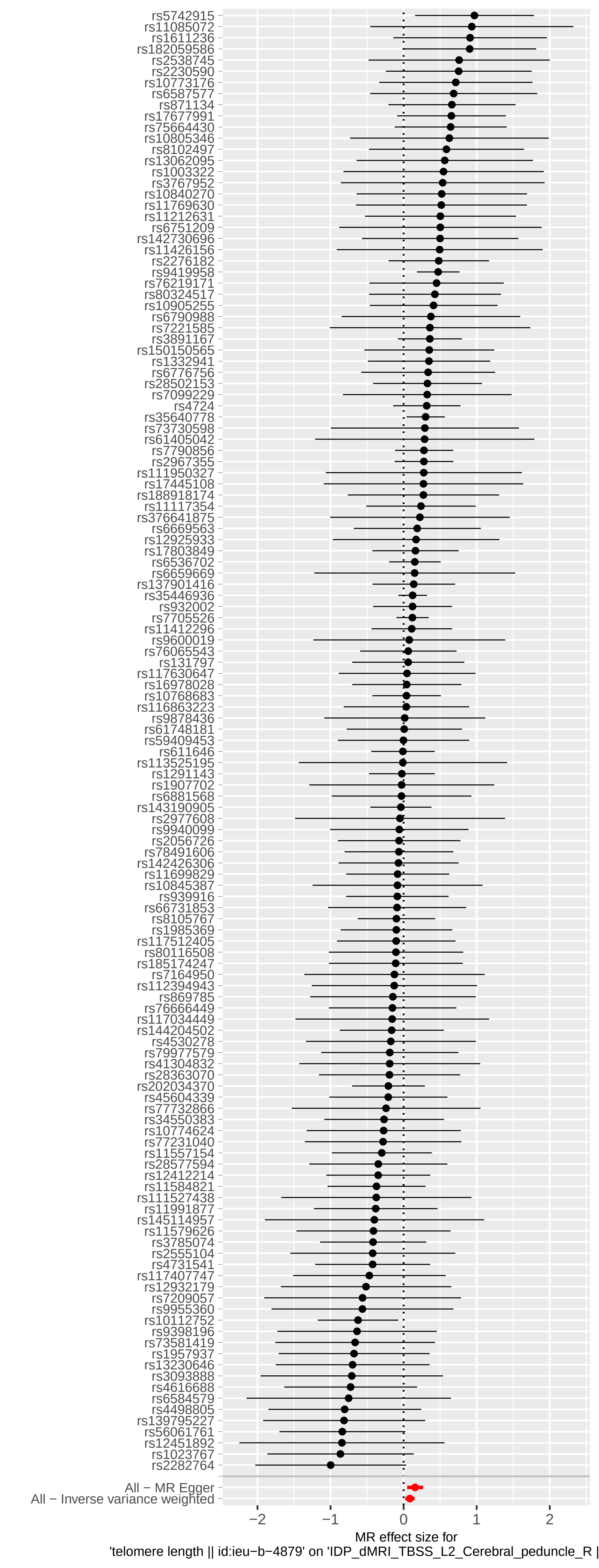
rs5742915
rs11085072
rs1611236
rs182059586
rs2538745
rs2230590
rs10773176
rs6587577
rs871134
rs17677991
rs75664430
rs10805346
rs8102497
rs13062095
rs1003322
rs3767952
rs10840270
rs11769630
rs11212631
rs6751209
rs142730696
rs11426156
rs2276182
rs9419958
rs76219171
rs80324517
rs10905255
rs6790988
rs7221585
rs3891167
rs150150565
rs1332941
rs6776756
rs28502153
rs7099229
rs4724
rs35640778
rs73730598
rs61405042
rs7790856
rs2967355
rs111950327
rs17445108
rs188918174
rs11117354
rs376641875
rs6669563
rs12925933
rs17803849
rs6536702
rs6659669
rs137901416
rs35446936
rs932002
rs7705526
rs11412296
rs9600019
rs76065543
rs131797
rs117630647
rs16978028
rs10768683
rs116863223
rs9878436
rs61748181
rs59409453
rs611646
rs113525195
rs1291143
rs1907702
rs6881568
rs143190905
rs2977608
rs9940099
rs2056726
rs78491606
rs142426306
rs11699829
rs10845387
rs939916
rs66731853
rs8105767
rs1985369
rs117512405
rs80116508
rs185174247
rs7164950
rs112394943
rs869785
rs76666449
rs117034449
rs144204502
rs4530278
rs79977579
rs41304832
rs28363070
rs202034370
rs45604339
rs77732866
rs34550383
rs10774624
rs77231040
rs11557154
rs28577594
rs12412214
rs11584821
rs111527438
rs11991877
rs145114957
rs11579626
rs3785074
rs2555104
rs4731541
rs117407747
rs12932179
rs7209057
rs9955360
rs10112752
rs9398196
rs73581419
rs1957937
rs13230646
rs3093888
rs4616688
rs6584579
rs4498805
rs139795227
rs56061761
rs12451892
rs1023767
rs2282764
All − MR Egger
All − Inverse variance weighted
−2
−1
0
1
2
MR effect size for
'telomere length || id:ieu−b−4879' on 'IDP_dMRI_TBSS_L2_Cerebral_peduncle_R |
